# Hollow manganese dioxide-chitosan hydrogel for the treatment of atopic dermatitis through inflammation-suppression and ROS scavenging

**DOI:** 10.1186/s12951-023-02174-w

**Published:** 2023-11-17

**Authors:** Yaguang Wu, Zihao Zhou, Min Zhang, Song Li, Mengyi Sun, Zhiqiang Song

**Affiliations:** 1 Department of Dermatology, Southwest Hospital, Third Military Medical University (Army Medical University), St 30 Gaotanyan, Chongqing, 400038 China; 2 Department of Rehabilitation, The Second Affiliated Hospital of Xinjiang Medical University, North 2nd Lane, Urumqi, 830000 China

**Keywords:** Atopic dermatitis, Hydrogel, Manganese dioxide, Reactive oxygen species

## Abstract

Atopic dermatitis (AD) is a chronic inflammatory disease associated with immune dysfunction. High levels of reactive oxygen species (ROS) can lead to oxidative stress, release of pro-inflammatory cytokines, and T-cell differentiation, thereby promoting the onset and worsening of AD. In this study, we innovatively used quaternary ammonium chitosan (QCS) and tannic acid (TA) as raw materials to design and prepare a therapeutic hydrogel(H-MnO_2_-Gel) loaded with hollow manganese dioxide nanoparticles (H-MnO_2_ NPs). In this system, the hydrogel is mainly cross-linked by dynamic ion and hydrogen bonding between QCS and TA, resulting in excellent moisture retention properties. Moreover, due to the inherent antioxidant properties of QCS/TA, as well as the outstanding H_2_O_2_ scavenging ability of H-MnO_2_ NPs, the hydrogel exhibits significant ROS scavenging capability. In vitro experiments have shown that H-MnO_2_-Gel exhibits good cellular biocompatibility. Importantly, in an AD-induced mouse model, H-MnO_2_-Gel significantly enhanced therapeutic effects by reducing epidermal thickness, mast cell number, and IgE antibodies. These findings suggest that H-MnO_2_-Gel, by effectively clearing ROS and regulating the inflammatory microenvironment, provides a promising approach for the treatment of AD.

## Introduction

Atopic dermatitis (AD) is a chronic allergic inflammatory skin disease that affects approximately 10% of adults and 20% of children worldwide. Its main features include dry skin, eczema, itching, and an excessive reaction to environmental stimuli [1, 2]. Over the past few decades, the prevalence and incidence of AD have increased due to exposure to environmental irritants such as air pollution and household cleaning products. Steroids, antihistamines, and antibiotics are commonly used to treat AD, however, they can cause long-term adverse effects such as hyperglycemia, poor wound healing, Cushing’s syndrome, and sleep disorders [3, 4]. As a result, research on more effective AD treatments for clinical use has become a top priority.

In recent years, an increasing number of studies have shown that elevated oxidative stress is closely related to the development of reactive oxygen species (ROS) in AD [5–7]. ROS include hydrogen peroxide (H_2_O_2_), superoxide anion (O_2_·^−^), and hydroxyl radicals (·OH), which are by-products of cellular metabolism. At normal levels, ROS act as important second messengers mediating cellular responses, thereby activating immune cells [8]. However, excessive ROS can cause high oxidative stress, promoting fatal oxidative damage to DNA and proteins and lipid peroxidation in AD patients, leading to cell death and aggravating AD conditions [9]. In addition, ROS are involved in signaling pathways such as NF-κB and p38 MAPK, leading to an increase in related pro-inflammatory cytokines, inducing additional T-cell differentiation and macrophage polarization, which are related to the development and worsening of AD [10, 11]. Thus, inhibiting oxidative stress induced by ROS in AD lesions may be a potential strategy for treating AD.

Hollow manganese dioxide nanoparticles (H-MnO_2_ NPs), as a type of inorganic nanoparticles, have the ability to efficiently scavenge ROS, mainly in the forms of MnO_2_, Mn_2_O_3_, and Mn_3_O_4_ [12]. Among these three forms, MnO_2_ exhibits the best catalytic properties [13]. MnO_2_ NPs usually break into Mn^2+^ and decompose H_2_O_2_ to O_2_ when reacting with H_2_O_2_. On the other hand, Mn^2+^ can eliminate O_2_·^−^ through cyclic redox reactions [14]. Due to its excellent properties, H-MnO_2_ NPs holds great potential in various environmental applications. Previous studies have reported the applications of H-MnO_2_ NPs in areas such as tumor therapy [15], drug delivery [16], and osteoarthritis treatment [17]. Furthermore, as a H_2_O_2_ scavenger, H-MnO_2_ NPs possess the ability to eliminate excessive ROS, making them highly promising in the field of inorganic materials [18]. Additionally, H-MnO_2_ NPs exhibit a dual role in promoting skin wound healing, as they can act on ROS while also generating localized oxygen. Compared to solid MnO_2_ NPs, H-MnO_2_ NPs have a higher specific surface area. Additionally, due to their porosity, reactants can more easily enter and exit the MnO_2_ NPs, resulting in a higher reaction rate [19]. Yang et al. developed an intelligent biodegradable H-MnO_2_ nanocoating that can modulate the hypoxic tumor microenvironment to enhance cancer therapy, thereby generating a synergistic effect favorable for anti-tumor immune response [15].

Hydrogels have a complex network structure and are highly hydrophilic biomaterials, widely used as drug carriers for various diseases [20]. The incorporation of functional nanomaterials has given hydrogel systems a broad range of applicability. Recently, various nanomaterials with unique ROS-regulating capabilities have been explored to guide the dynamic behavior of ROS in pathological microenvironments and alleviate inflammation. Kim et al. developed a hydrogel embedded with CeO_2_ nanoparticles that can effectively eliminate ROS. This hydrogel leads to a reduction of extracellular and intracellular ROS and exhibits cellular protective effects under highly oxidative conditions [21].

**Scheme 1:**
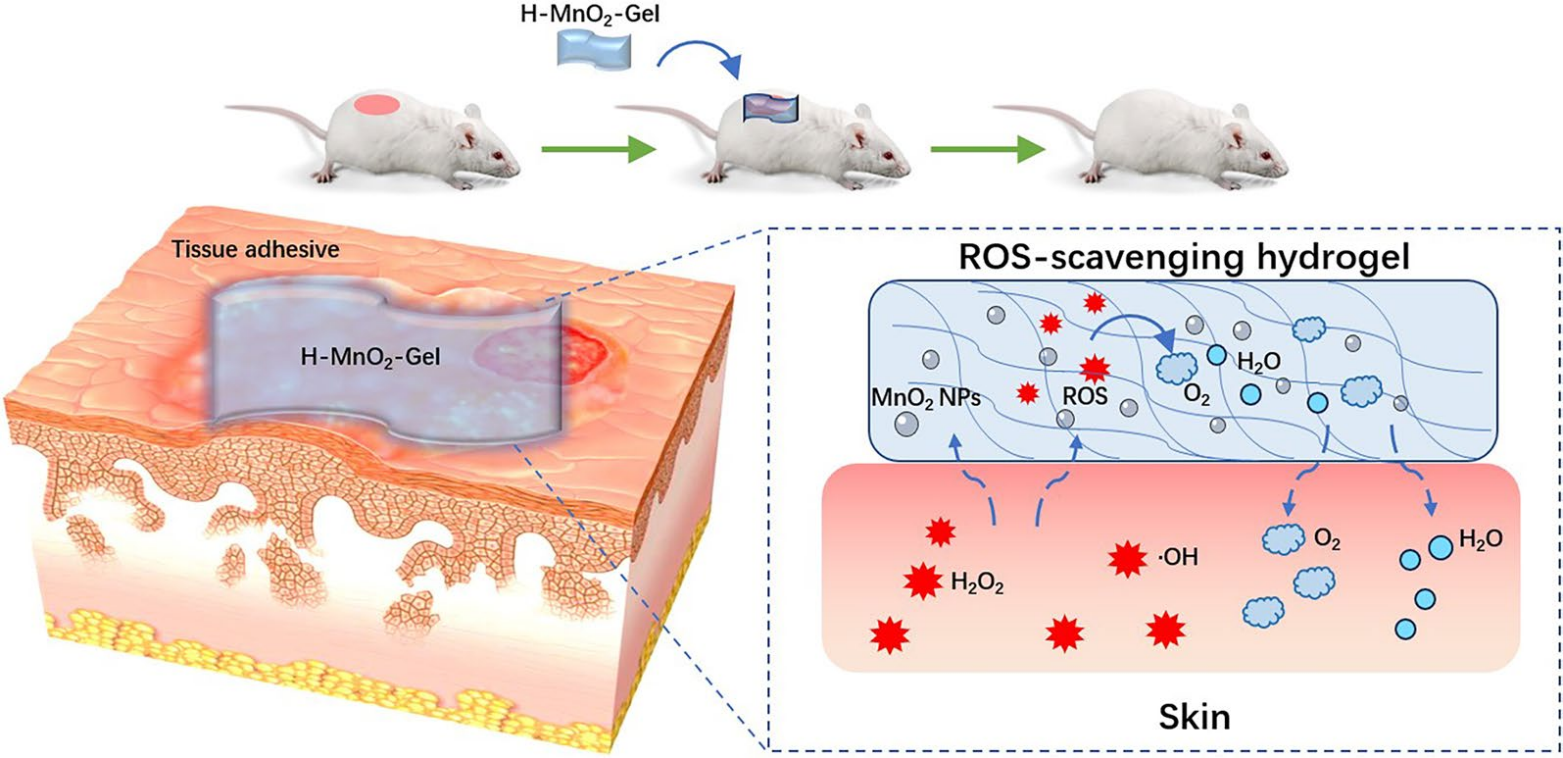
Schematic of the H-MnO_2_-Gel, which suppresses oxidative stress and decreases inflammatory response

Here, we report a therapeutic hydrogel for the treatment of AD by scavenging ROS (Scheme 1), which includes quaternary ammonium chitosan (QCS)/tannic acid (TA) hydrogel embedded with H-MnO_2_ NPs. TA possesses advantages such as antioxidant, anti-inflammatory, and biodegradable properties. Ahmadian et al. have demonstrated that TA hydrogel can effectively remove excessive ROS in wounds and promote wound healing [22]. We hypothesize that, upon generating highly abundant ROS in the dermis of mice, ROS-scavenging therapeutic H-MnO_2_@QCS/TA hydrogels (H-MnO_2_-Gel) can be used as a reservoir to store H-MnO_2_ NPs and TA. After application to the skin site, they can work synergistically to effectively scavenge ROS. In this work, H-MnO_2_-Gel exhibit good mechanical properties and biocompatibility, with efficient capabilities to scavenge ROS by simulating catalase, as well as providing cell protection under 1-chloro-2,4-dinitrobenzene (DNCB)-induced high oxidative stress. In animal experiments using direct skin exposure to DNCB sensitization-induced AD models, the application of H-MnO_2_-Gel to AD skin lesions demonstrated therapeutic effects, characterized by reduced epidermal thickness, decreased accumulation of DNA oxidative damage products, and reduced Th2 cytokines, IgE, and tissue-infiltrating mast cells. These findings suggest that this novel ROS-scavenging hydrogels provides a valuable approach for the treatment of AD.

## Methods and materials

### Materials

The following materials were acquired and used in the study: potassium permanganate (Merck), SiO_2_ (Ruixi), quaternary ammonium chitosan (QCS, Ruixi), tannic acid (TA, Ruixi), Triton X-100 (Aladdin), polydopamine (Ruixi), tetraethyl orthosilicate (TEOS, Merck), cyclohexane (Merck), n-Hexanol (Merck), catalase (CAT) assay kit (ammonium molybdate colorimetric method) (Bestbio), safranin O solution (Solarbio), free DPPH radical scavenging capacity assay kit(Solarbio), RPMI-1640 cell culture medium (Gibco), fetal bovine serum (FBS, BI), olive oil (Sigma-Aldrich), acetone (Daejung), 2′,7′-dichlorofluorescin diacetate (DCFDA) cellular ros assay kit (Abcam), MTT cell proliferation and cytotoxicity assay kit (Beyotime), dimethyl sulfoxide (DMSO, Beyotime), 1-chloro-2,4-dinitrobenzene (DNCB, Merck), 4% formaldehyde (Biosharp), methylene blue (Sigma-Aldrich), mouse TSLP ELISA kit (Arigo), mouse IgE ELISA kit (Beyotime), mouse IL-4 ELISA kit (Beyotime), mouse IL-10 ELISA kit (Beyotime).

### Synthesis of H-MnO_2_ NPs

The H-MnO_2_NPs were synthesized according to previous literature[23]. In brief, solid silica NPs (sSiO_2_) were first synthesized as templates. Triton X-100 (53 mL), cyclohexane (225 mL), and n-Hexanol (54 mL) were mixed and stirred for 1 h. Then, ammonia (7.5 mL), water (10 mL), and TEOS (5 mL) were immediately added, and the mixed solution was stirred for 12 h. The SiO_2_ NPs were collected by centrifugation and repeated washing. 123 mg of SiO_2_ and 100 mg of polydopamine (PDA) were stirred in 50 mL of Tris buffer (10 mM, pH 8.5) at room temperature for 3 h, and SiO_2_@PDA was collected by centrifugation. Then, a KMnO_4_ aqueous solution (2.5 mg mL_−1_, 40 mL) was added dropwise, and the reaction continued stirring for 6 h. The resulting NPs were collected by centrifugation and then etched with a 1 M NaOH solution at 80℃ for 6 h to obtain the H-MnO_2_ NPs.

### Synthesis of QCS/TA hydrogel and H-MnO_2_-gel

Firstly, 3% (w/v) QCS solution and 5% TA solution were prepared by dissolving QCS and TA powders in deionized water, respectively. Then, equal volumes of QCS solution and TA solution were simply mixed to obtain QCS/TA hydrogels containing 1.5% QCS and 2.5% TA. To prepare H-MnO_2_-Gel, prepare a mixture of 5% TA solution and H-MnO_2_ NPs, then add 0.15 mL of HCl solution and add it to an equal volume of 3% (w/v) QCS solution. After mixing the above solution using ultrasound (PZ-550LI, Fangxu), mix it with a NaHCO_3_ solution in a ratio of 10:1. Rapidly stir the mixture for 30 s to prepare and obtain H-MnO_2_-Gel.

### Characterization methods

A suite of characterization methods was employed. Transmission electron microscopy (JEM-2100, JEOL) was used to observe the morphology of the H-MnO_2_ NPs. To confirm the successful loading of H-MnO_2_ NPs into the QCS/TA hydrogel, the content of H-MnO_2_ NPs in hydrogel was determined using Energy-dispersive X-ray spectroscopy (EDS) mapping. Fourier transform infrared (FT-IR) spectra were recorded using a Nicolet iS50 FTIR spectrometer (Thermo Scientific). Dynamic light scattering (DLS) (Malvern Zetasizer Nano-ZS90) was used to observe the nanoparticle size and zeta potential of the H-MnO_2_ NPs. The water content was calculated by subtracting the weight of the freeze-dried hydrogel from the weight of the hydrogel soaked overnight. For the compression test, the hydrogels were prepared as cylinders with a diameter of 10 mm and a height of 10 mm. The compression test was performed using a QC-508B1 (Cometech) compression testing machine at a speed of 70 mm/min and a load of 100 N, with a compression strain measurement range of up to 80%. To conduct the tensile test, the hydrogels were fabricated into a dumbbell shape with specific dimensions: a narrow width of 15 mm, narrow length of 10 mm, broad width of 50 mm, and overall length of 70 mm. All measurements were conducted with a minimum of three repetitions.

### The catalytic activity measurement of H-MnO_2_NPs and H-MnO_2_ gel

The catalytic activity of H-MnO_2_ NPs was determined using an ammonium molybdate colorimetric assay. In brief, mix 20 µL of different concentrations (0 µg/mL, 50 µg/mL, 100 µg/mL, 200 µg/mL) of H-MnO_2_ NPs solution with 45 µL of different concentrations (25 µM, 50 µM, 100 µM) of H_2_O_2_ and 85 µL of PBS, and incubate at 25 ℃ for 60 min. Subsequently, 50 µL of 250 mM ammonium molybdate solution was added to the reaction mixture to terminate the catalytic reaction. The nanoparticles were removed by centrifugation at 10,000 rpm for 5 min. The absorbance of the supernatant was measured at 405 nm. To determine the catalytic activity of the hydrogel, Blank-Gel and H-MnO_2_ Gel with an area of 1 cm^2^ and a thickness of 1 mm were added to each well of a 12-well plate. Then, 1 mL of 20 µM H_2_O_2_ solution was added to each well. After incubation on a shaker, at fixed time points (0 min, 10 min, 20 min, 30 min, 40 min, 50 min, 60 min), the solution was collected. Next, 350 µL of 250 mM ammonium molybdate solution was added to the reaction mixture to terminate the catalytic reaction. The absorbance of the supernatant was measured at 405 nm. According to the kinetic curve based on the Michaelis-Menten equation, the Michaelis-Menten constant (K_m_) was calculated using the equation:

$$v = {{v\max \left[ S \right]} \mathord{\left/ {\vphantom {{v\max \left[ S \right]} {\left( {Km + \left[ S \right]} \right)}}} \right. \kern-\nulldelimiterspace} {\left( {Km + \left[ S \right]} \right)}}$$

In this equation, v represents the initial velocity, v_max_ represents the maximum catalytic velocity, [S] represents the substrate concentration, and K_m_ represents the Michaelis constant.

### Antioxidant property of hydrogels

Safranin O assay: Mix 300 µL of hydrogel with 600 µL of 2 mM ferrous sulfate solution and 500 µL of 360 µg/mL Safranin O solution, and incubate for 10 min. Then, add 800 µL of 6% H_2_O_2_ solution and incubate at 55 ℃ for 60 min. For the blank group, use 300 µL of deionized water instead of hydrogel. For the control group, use 300 µL and 800 µL of deionized water instead of hydrogel and H_2_O_2_ solution, respectively. Measure the absorbance of the mixture at 492 nm using a microplate reader. Calculate the scavenging ability of the hydrogel against ·OH using the following formula:

$${\text{Savenging}}{\mkern 1mu} {\,\text{ability}}{\mkern 1mu} (\% ) = {{\left( {{\text{A}}_{{{\text{Hyd}}}} - {\text{A}}_{{{\text{Blan}}}} } \right)} \mathord{\left/ {\vphantom {{\left( {{\text{A}}_{{{\text{Hyd}}}} - {\text{A}}_{{{\text{Blan}}}} } \right)} {\left( {{\text{A}}_{{{\text{Con}}}} - {\text{A}}_{{{\text{Blan}}}} } \right)}}} \right. \kern-\nulldelimiterspace} {\left( {{\text{A}}_{{{\text{Con}}}} - {\text{A}}_{{{\text{Blan}}}} } \right)}} \times 100\%$$

DPPH assay: To evaluate the hydrogel’s ability to scavenge DPPH radicals, a 300 µL hydrogel sample was prepared in a 24-well plate and immersed in 1 mL of anhydrous ethanol. Following that, 100 µL of DPPH solution (dissolved in 0.5 mM ethanol) was added, and the mixture was incubated for 60 min in darkness. In the control group, 300 µL of deionized water was used instead of the hydrogel. The absorbance of the reaction mixture was measured at 517 nm, and the hydrogel’s scavenging efficiency of DPPH radicals was calculated using the equation:

$${\text{Savenging}}{\mkern 1mu} {\,\text{efficiency}}{\mkern 1mu} (\% ) = {{\left( {{\text{A}}_{{{\text{Con}}}} - {\text{A}}_{{{\text{Hyd}}}} } \right)} \mathord{\left/ {\vphantom {{\left( {{\text{A}}_{{{\text{Con}}}} - {\text{A}}_{{{\text{Hyd}}}} } \right)} {{\text{A}}_{{{\text{Con}}}} \times 100\% }}} \right. \kern-\nulldelimiterspace} {{\text{A}}_{{{\text{Con}}}} \times 100\% }}$$

### In vitro cytotoxicity and cytoprotective experiments of blank-gel and H-MnO_2_-gel under high oxygen conditions

The mouse fibroblast cell line (L929) was purchased from the Cell Bank of the Chinese Academy of Sciences. The cell culture was conducted using RPMI-1640 medium supplemented with 10% FBS and 1% penicillin/streptomycin, as recommended. For the in vitro cytotoxicity assay, after 24 h of cell culture, the old culture medium was removed. H-MnO_2_-Gel (approximately 5 mm in diameter and 1 mm in thickness) was placed on the surface, and an equal amount of RPMI-1640 complete medium was added as a blank control. After culturing under conditions of 37 ℃ and 5% CO_2_ for 24 h, H-MnO_2_-Gel was removed, and the cells were stained with calcein-AM and propidium iodide. Cell viability was observed using fluorescence microscopy. After 24 and 48 h of culture, the cell viability at 570 nm was determined using the tetrazolium blue colorimetric assay.

In the H_2_O_2_ cell protection assay, L929 cells with a density of 5 × 10^3^ were seeded in a 96-well plate. After 24 h, when Blank-Gel and H-MnO_2_-Gel were present, 200 µL of RPMI1640 medium containing 1000 µM H_2_O_2_ was added. After 24 h, the cell viability was determined using the methyl thiazolyl tetrazolium (MTT) assay. In the MTT assay, L929 cells in logarithmic growth phase were seeded into a 96-well plate. After that, they were treated with 10 µL of tetrazolium salt solution. The cells were then incubated for 4 h, and the supernatant was removed. Subsequently, 100 µL of dimethyl sulfoxide (DMSO) was added as a solubilizing and lysing solution. Finally, the optical density at a wavelength of 570 nm was measured.

In the cytoprotective test using DNCB, L929 cells were cultured in a 6-well plate with a density of 5 × 10^5^ cells per well and incubated overnight. Subsequently, 60 µM of DNCB was added to each well. After incubating for 30 min, the cells were washed and the medium was replaced. The cells were then incubated with hydrogels for 2 h. In the control group, the cells were incubated in fresh medium for 2 h. The cells were then resuspended in PBS and transferred to a 96-well plate. The cells were stained with 5 µM DCFDA solution and incubated for 30 min. The stained cells were analyzed using a flow cytometer.

### The therapeutic effect of H-MnO_2_-gel on the in vivo model of AD induced by DNCB

The experiment utilized 6-week-old female BALB/c mice as an in vivo model. The mice were randomly divided into 4 groups, with 6 mice in each group. All animal experimental procedures were approved by the Experimental Animal Center of the Army Medical University. On the day before the experiment, the dorsal fur of all mice was shaved to an area of approximately 4 cm². DNCB was used to induce AD on the dorsal skin of the mice, by dissolving a certain amount of DNCB in a 3:1 mixture of acetone and olive oil. In the 1st week, each mouse’s dorsal skin was topically applied with 120 µL of 0.2% (v/v) DNCB solution. DNCB application was then discontinued in the 2nd week, and in the 3rd week, the mice’s dorsal skin was sensitized by continuing with the application of 120 µL of 1% (v/v) DNCB solution, twice every other day, until the end of the 3rd week. In the fourth and 5th weeks, Blank-Gel and H-MnO_2_-Gel were applied once every other day.

Throughout the research process, we utilized an eczema scoring system to evaluate the severity of four typical symptoms of atopic dermatitis: erythema/bleeding, scaling/dryness, edema, and excoriation/erosion. Each symptom was graded on a scale of 0 (none), 1 (mild), 2 (moderate), or 3 (severe), and the total score was calculated as the sum of these individual scores, representing the eczema severity. At the conclusion of the study, all mice were euthanized in a humane manner. Dorsal skin tissue sections from the middle segment of the mice were collected and fixed with formalin solution for histological analysis. Following deparaffinization and rehydration, the sections were stained with hematoxylin and eosin (H&E) kit. Additionally, the sections were stained with DAPI. Finally, the stained sections were visualized under a microscope.

The epidermal thickness in H&E-stained images was measured using Image J software. Toluidine blue staining was utilized to count the number of mast cells in the images. Blood samples were collected from the hearts of the mice during euthanasia. Serum was separated and stored at − 80 ℃ before use. Enzyme-linked immunosorbent assay (ELISA) with sandwich double-antibody was employed to detect the levels of IgE, TSLP, IL-4, and IL-10 in the serum. All experiments were conducted following the instructions provided by the manufacturer.

### Statistical analysis

All results are presented as mean ± standard deviation. Non-paired, two-tailed Student’s t-tests were used to analyze the differences between any two groups. One-way ANOVA was employed to assess differences among more than two groups. Statistical significance was set at a P-value of < 0.05. GraphPad Prism 8.0 software was used for statistical analyses.

## Results and discussion

### Preparation and characterization of H-MnO_2_ NPs

**Fig. 1:**
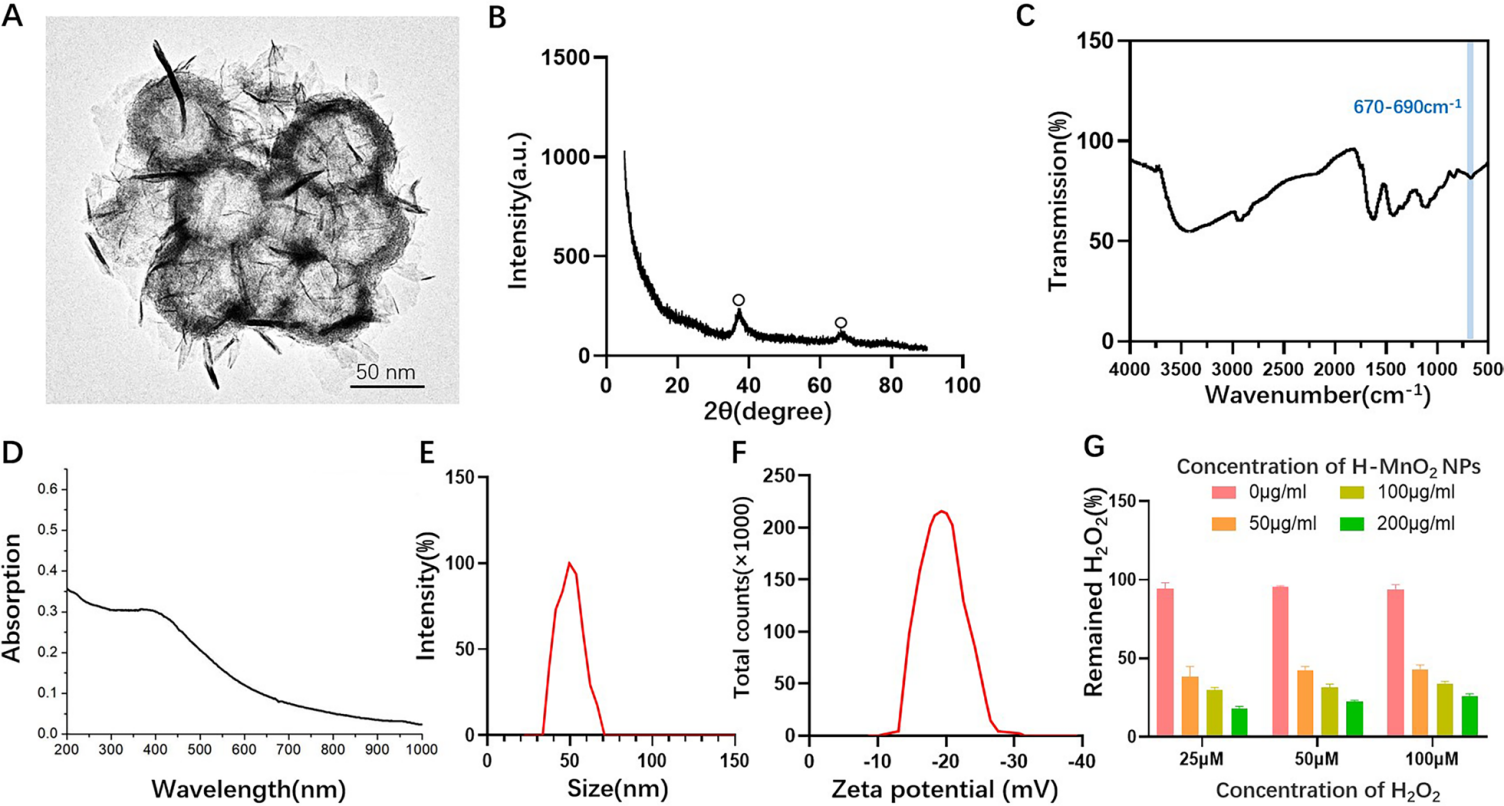
**A** TEM visualization of H-MnO_2_ NPs (scale bar = 50 nm). **B** X-ray diffraction pattern of H-MnO_2_ NPs. **C** FT-IR of H-MnO_2_ NPs. **D** UV-Vis absorption spectrum of H-MnO_2_ NPs. **E** Hydrodynamic size of H-MnO_2_ NPs. **F** Zeta potential of H-MnO_2_ NPs. **G** Catalase-mimicking activity of H-MnO_2_ NPs at various concentrations was determined using the colorimetric method with ammonium molybdate

By using SiO_2_ and KMnO_4_ as raw materials, dispersible H-MnO_2_ NPs were synthesized through etching with NaOH solution at 80 ℃. The diameter of H-MnO_2_ NPs can generally be controlled based on the specific synthesis method and conditions. Typically, the diameter of H-MnO_2_ NPs falls within the range of several nanometers to several tens of nanometers. The specific diameter depends on factors such as the template, solvent, reaction time, and temperature used in the synthesis process [24]. Uniform H-MnO_2_ NPs with a diameter of approximately 50 nm were observed using transmission electron microscopy (TEM)(Fig. 1A). X-ray diffraction (XRD) analysis showed the data of the two diffraction peaks displayed in Fig. 1B (2θ = 37.82°, 2θ = 66.54°) matched well with the pattern described in the α-MnO_2_ (PDF standard card #30–0820). As shown in Fig. 1C, the FT-IR spectrum of H-MnO_2_ NPs exhibits a stretching absorption peak of Mn-O located at 670–690 cm^−1^. Additionally, the results of the UV-Vis absorption spectroscopy indicate a broad absorption peak of H-MnO_2_ in the range of 240 to 600 nm (Fig. 1D). Dynamic light scattering (DLS) measurements revealed that the hydrodynamic size of H-MnO_2_ NPs was approximately 49.4 ± 0.8 nm with a PDI value of 0.11, indicating good control over the size distribution of the nanoparticles (Fig. 1E), and the Zeta potential was − 18.11 ± 1.01 mV (Fig. 1F). These results collectively demonstrate the successful preparation of H-MnO_2_ NPs.

In AD patients, the oxidative stress state is significantly increased in the skin, including an elevated production of H_2_O_2_. H_2_O_2_ is a ROS that can cause cellular damage and trigger inflammatory responses. In the skin affected by AD lesions, various cell types, such as inflammatory cells and keratinocytes, release H_2_O_2_, leading to an elevated local level of H_2_O_2_. To test the ROS scavenging performance of H-MnO_2_ NPs, we conducted in vitro experiments using different concentrations (25 µM, 50 µM, 100 µM) of H_2_O_2_ to evaluate the catalytic ability of different concentrations of H-MnO_2_ NPs. As shown in Fig. 1G, H-MnO_2_ NPs exhibited significant catalytic activity in the decomposition of H_2_O_2_ with the remaining amount of H_2_O_2_ in the 50 µg H-MnO_2_ NPs group approximately 1.65 times higher than that in the 200 µg H-MnO_2_ NPs group. This finding suggests that H-MnO_2_ NPs may react with high concentrations of H_2_O_2_ in AD to generate oxygen, thereby significantly reducing the H_2_O_2_ levels.

### Preparation and characterization of H-MnO_2_-gel

**Fig. 2:**
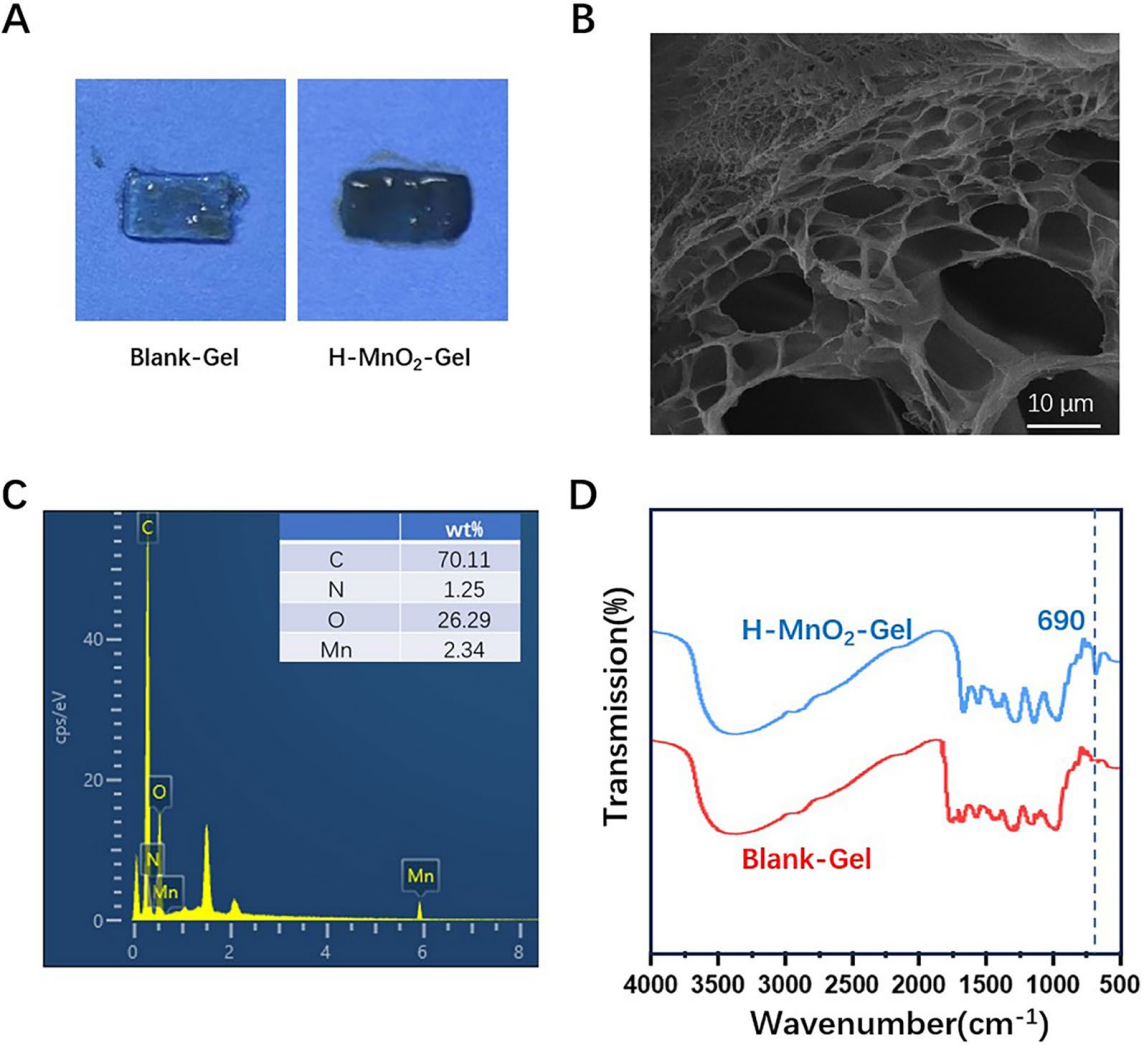
**A** The photo of the Blank-Gel and H-MnO_2_-Gel patches. **B** SEM image of cross section of freeze-dried H-MnO_2_-Gel. **C** EDS analysis of H-MnO_2_-Gel. **D** FT-IR spectra of Blank-Gel and H-MnO_2_-Gel

In this study, a simple physical crosslinking method was developed to prepare a QCS/TA hydrogel with antioxidative properties. This method involved the simple mixture of QCS and TA. The QCS/TA hydrogel (Blank-Gel) is transparent in the visible light wavelength range, and with the loading of H-MnO_2_ NPs, the transparency of the hydrogel decreases, resulting in a black appearance (Fig. 2A). The cross-section of the freeze-dried hydrogel was examined using scanning electron microscopy (SEM) to study the microstructure of the hydrogel. The results show that H-MnO_2_-Gel exhibits a porous structure with an interconnected network, with a dense distribution of pores ranging in size from 10 to 50 μm (Fig. 2B). X-ray energy dispersive spectroscopy (EDS) analysis reveals that the content of Mn in the freeze-dried hydrogel is approximately 2.34 wt% (Fig. 2C). FT-IR analysis was performed on H-MnO_2_-Gel, from Fig. 2D, it can be observed that H-MnO_2_-Gel exhibits a characteristic peak at 690 cm^−1^ in the FT-IR spectrum, indicating the presence of Mn-O bonds in the hydrogel.

Next, compression and tension tests were further conducted on the Blank-Gel and H-MnO_2_-Gel. After the addition of H-MnO_2_ NPs, H-MnO_2_-Gel exhibited similar elastic modulus to Blank-Gel in compression and tension tests. The water content of both Blank-Gel and H-MnO_2_-Gel was approximately 95% (Table 1). In conclusion, our work has confirmed the synthesis of hydrogels and the successful loading of H-MnO_2_ NPs. The hydrogels loaded with H-MnO_2_ exhibit similar mechanical properties and high water content as QCS/TA hydrogels.

**Table 1:** Elastic modulus assessed by compression and tensile tests and water content of Blank-Gel and H-MnO_2_-Gel

	Blank-gel	H-MnO_2_-gel	P value
Compression test elastic modulus (kPa)	6.23 ± 1.12	6.10 ± 0.97	0.52
Tensile tests elastic modulus (kPa)	13.10 ± 0.85	12.82 ± 1.44	0.79
Water contents(%)	95.16 ± 0.77	94.84 ± 0.65	0.37

### Antioxidative property of blank-gel and H-MnO_2_-gel

**Fig. 3:**
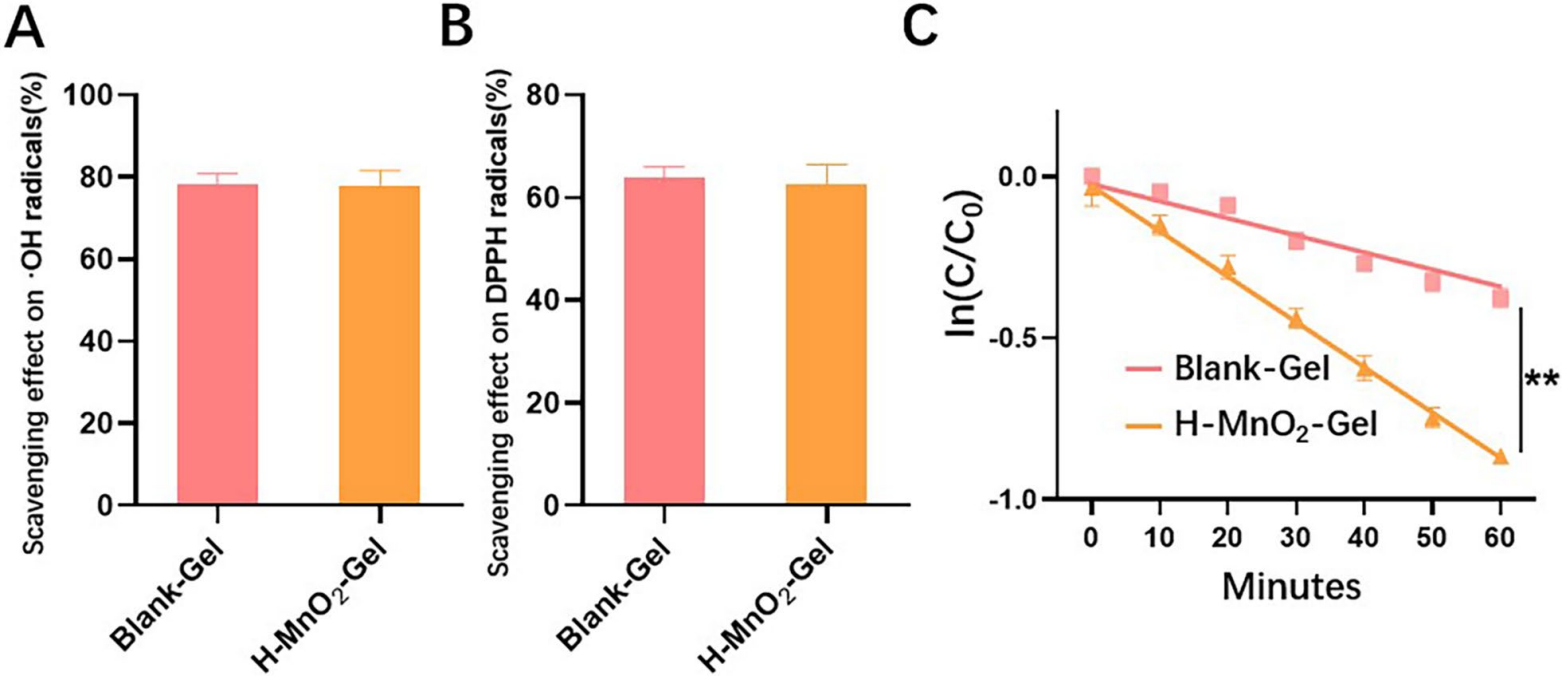
The scavenging ability of Blank-Gel and H-MnO_2_-Gelusing (**A**) Safranin O assay for hydroxyl radicals, and (**B**)DPPH assay for DPPH radicals. (**C**) ln(C/C_0_)–time course curves of H_2_O_2_ decomposition catalyzed by Blank-Gel and H-MnO_2_-Gel. **P<0.01

Low levels of ROS are beneficial for the recovery of AD, while excessive ROS can lead to severe inflammation, thus negatively affecting AD. QCS/TA hydrogel (Blank-Gel) has strong ROS scavenging ability and plays a positive role in the recovery of AD. The antioxidant activity of Blank-Gel and H-MnO_2_-Gel was evaluated through scavenging experiments of hydroxyl radicals and DPPH radicals. As shown in Fig. 3A and B, all hydrogel groups exhibited strong scavenging abilities for hydroxyl radicals (> 75%) and DPPH radicals (> 64%) as detected by Safranin O and DPPH assays, but there was no significant difference between Blank-Gel and H-MnO_2_-Gel. These data indicate that Blank-Gel itself has the potential to capture free radicals and possesses good antioxidant capacity. Oxidative stress caused by free radicals often damages surrounding tissue cells and prolongs inflammation process. We used 20 µM H_2_O_2_ to simulate the H_2_O_2_ concentration in the microenvironment of AD dermal tissue and conducted in vitro tests on the catalytic activity of Blank-Gel and H-MnO_2_-Gel. Compared to Blank-Gel, the reaction solution of H-MnO_2_-Gel showed significantly enhanced catalytic activity towards H_2_O_2_. As shown in Fig. 3C, the rate constant for H-MnO_2_-Gel in decomposing H_2_O_2_ was 2.1 times higher than that of Blank-Gel. This indicates that the efficient H_2_O_2_ decomposition ability of H-MnO_2_ NPs enhances the intrinsic antioxidant capacity of Blank-Gel, and their combination can efficiently reduce ROS levels. These data suggest H-MnO_2_-Gel could effectively eliminate ROS generated within cells, thereby protecting cells from oxidative stress damage during the recovery process of AD.

### Biocompatibility of H-MnO_2_-gel

**Fig. 4:**
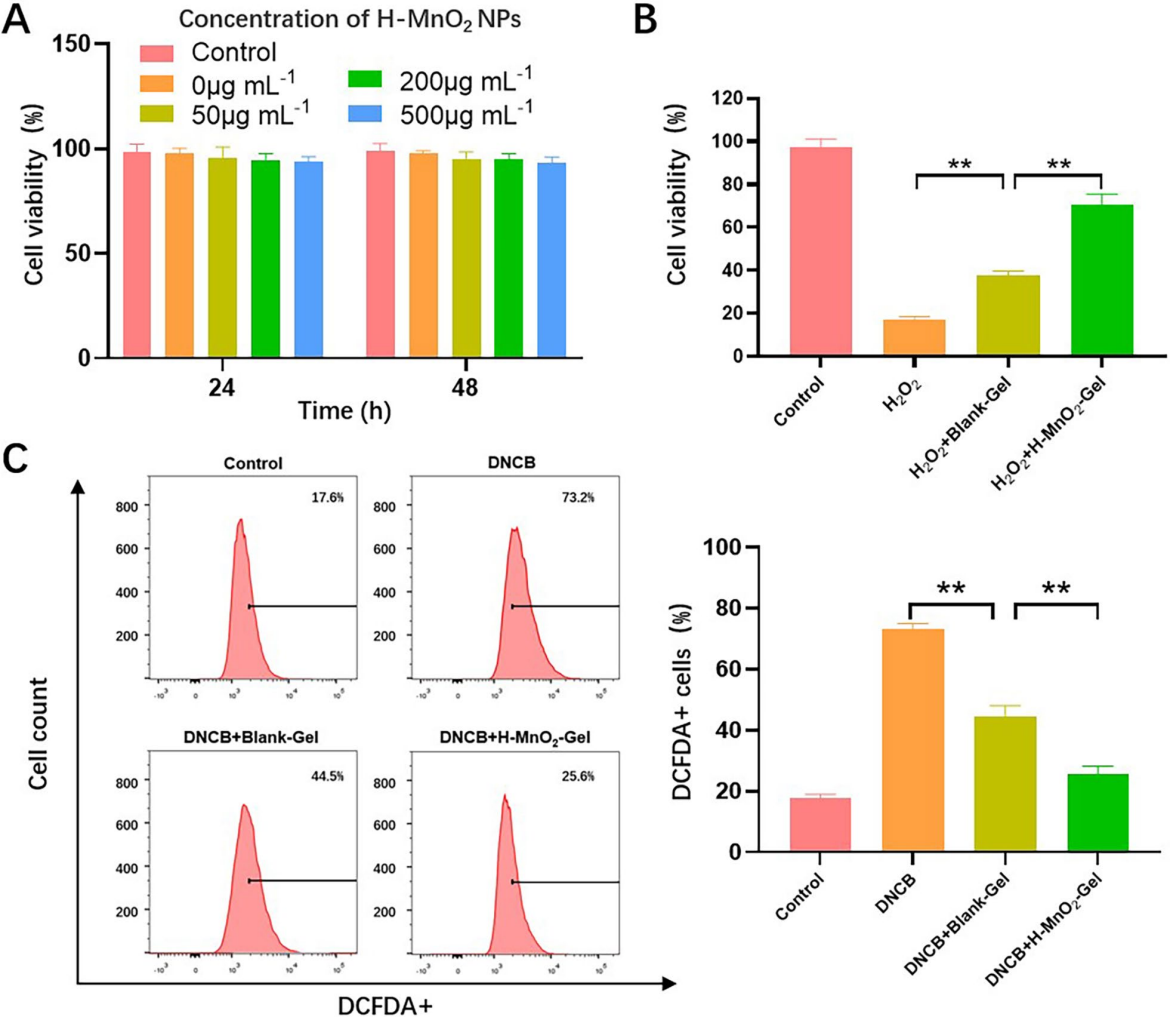
**A** Cell viability of L929 cells treated with H-MnO_2_-Gel at different concentrations of H-MnO_2_ NPs at 24 and 48 h. **B** Cytoprotective effect of H-MnO_2_-Gel under a highly oxidative medium (1000 µM H_2_O_2_). **C** Flow cytometry analysis and quantitative analysis of intracellular ROS levels of DNCB-treated cells after incubating with Blank-Gel and H-MnO_2_-Gel. **P < 0.01

In AD patients, fibroblasts have been reported to affect the differentiation and proliferation of keratinocytes [25]. To further evaluate the potential cytotoxic effects of H-MnO_2_-Gel, we co-cultured mouse fibroblast L929 cells with hydrogel containing different concentrations of H-MnO_2_ NPs, and assessed the cytotoxicity of H-MnO_2_-Gel. The MTT assay is employed to assess the viability of cells. As shown in Fig. 4A, the OD values were measured at 570 nm utilizing the MTT assay. Even when the concentration of H-MnO_2_ NPs exceeded 500 µg/mL, the proportion of viable cells did not significantly decrease. Through the testing, we have found that the cell viability of L929 cells is reduced to less than 20% when cultured in a medium containing 1000 µM H_2_O_2_. To evaluate the cell protective ability of H-MnO_2_-Gel in a highly oxidative environment, Blank-Gel or H-MnO_2_-Gel (with a diameter of 5 mm and a thickness of 1 mm) were simultaneously added to a culture medium containing 1000 µM H_2_O_2_. Compared to the Blank-Gel group, the addition of of H-MnO_2_ NPs to H-MnO_2_-Gel further significantly improved cell viability (Fig. 4B), indicating that H-MnO_2_ NPs can effectively prevent cell damage by removing excessive H_2_O_2_ from the culture medium in synergy with QCS/TA hydrogel.

Next, we demonstrated the cell protective effect of H-MnO_2_-Gel under conditions of high intracellular ROS generation. DNCB is a chemical used to prepare AD animal models, which increases cellular ROS levels by application to the skin. Mouse fibroblast L929 cells was incubated in DNCB (60 µM) for 30 min and thoroughly washed. Then, Blank-Gel or H-MnO_2_-Gel was added to the culture medium and incubated for another 2 h. Subsequently, Dichlorodihydrofluorescein diacetate (DCFDA) staining was used to detect the fluorescence of intracellular ROS. The experiment showed that DNCB-cultured L929 cells showed significantly elevated levels of intracellular ROS (Fig. 4C). When DNCB-treated cells were co-incubated with Blank-Gel or H-MnO_2_-Gel, intracellular ROS decreased by 28.7% and 47.9%. ROS can be transported both inside and outside cells through water channels on the cell membrane. In the case of DNCB treatment, excessive intracellular ROS may diffuse into the extracellular space. Comparing to the control group, Blank-Gel is capable of absorbing a portion of the diffused ROS, which aids in reducing intracellular ROS levels. On the other hand, H-MnO_2_-Gel not only absorbs the diffused ROS but also benefits from the presence of H-MnO_2_ NPs within the hydrogel. This component significantly enhances the decomposition rate of H_2_O_2_, thus facilitating continuous and efficient elimination of ROS. Consequently, the microenvironment experiences a significant reduction in ROS levels.

### The therapeutic effect of H-MnO_2_ -gel on the in vivo model of AD induced by DNCB

**Fig. 5:**
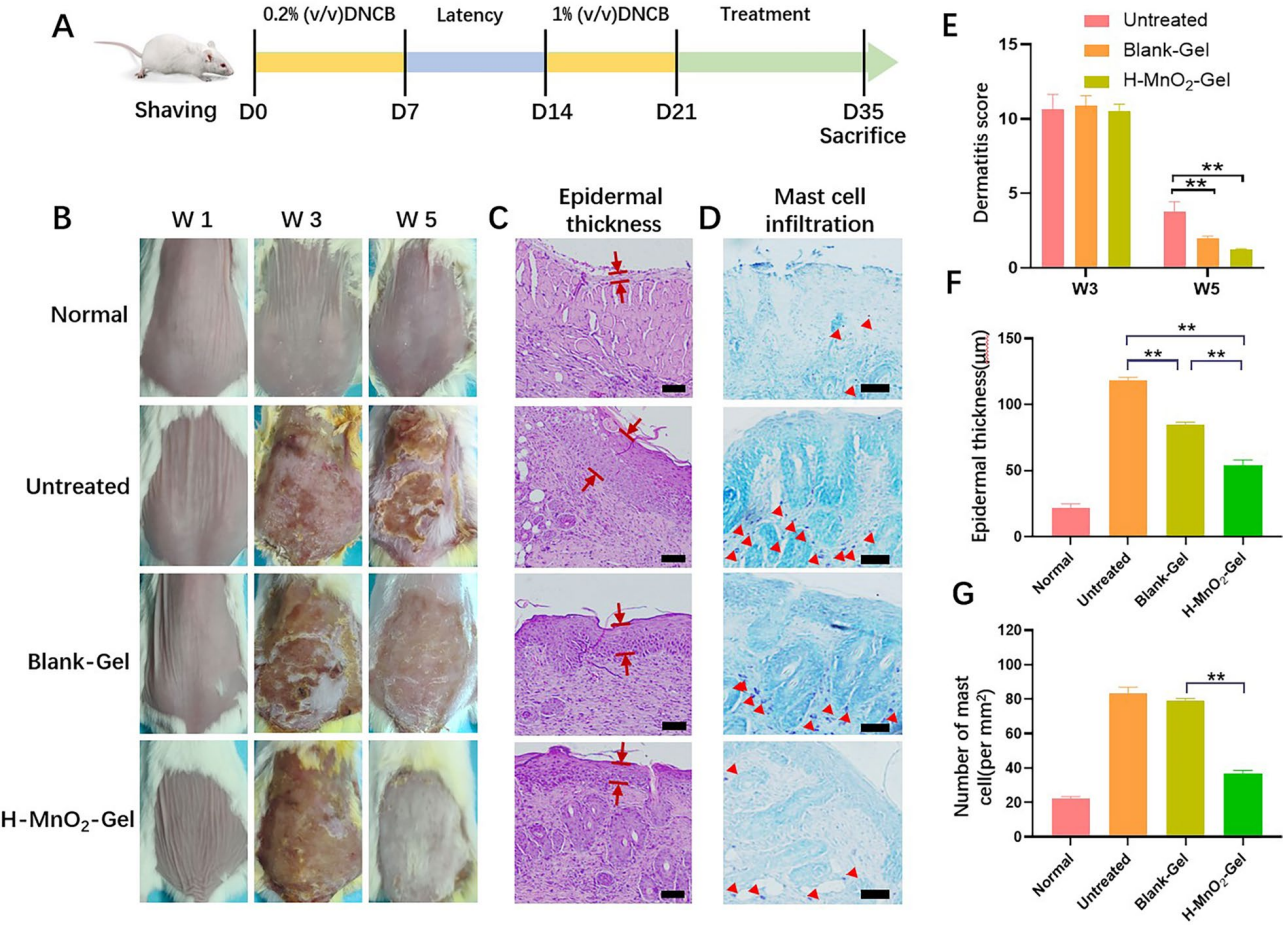
**A** Timeline for conducting in vivo experiments on AD induction through sensitization with DNCB and subsequent treatments using hydrogel patches. **B** Representative photographs of dorsal skin of each group for monitoring the change in the lesion. **C** Histological analysis of mouse skin tissue sections stained with H&E. The distance between the red lines represents the measurement of epidermal thickness. **D** Histological examination of mouse skin tissue sections stained with toluidine blue to identify dermal mast cells. The mast cells are indicated by red arrowheads (scale bar = 100 μm). **E** Dermatitis score evaluations performed over a duration of 5 weeks. **F** Comparison of epidermal thickness. **G** Quantification of mast cell count for each treatment group. (n = 6). **P < 0.01

Through the above experiments, it has been demonstrated that H-MnO_2_-Gel can significantly accelerate the recovery of AD by regulating oxidative stress. To investigate the potential of H-MnO_2_-Gel in treating AD, we induced a mouse AD model using DNCB. DNCB is one of the chemicals commonly used to create animal models of AD. When applied to the skin, DNCB interacts with skin proteins to form complexes, which are taken up by antigen-presenting cells and subsequently activate Th2 cells and mast cells [26]. During the first sensitization period, 0.2% DNCB was applied to the mouse’s dorsal skin three times a week for 1 week, followed by a one-week rest period. Then, 1% DNCB was applied for 7 days to induce IgE crosslinking and Th2-mediated immune dysfunction mimicking severe AD (Fig. 5A). As shown in Fig. 5B W3 of the DNCB-treated group, the skin of mice exhibited compounds containing blood and pus, indicating successful induction of AD in the skin during the 3rd week. From the 4th week onwards, for the following 14 days, mice either received no treatment or were treated with either Blank-Gel or H-MnO_2_-Gel. During the treatment period, gauze soaked in 0.2% DNCB was applied between hydrogel treatments to simulate continuous allergen exposure. After 14 days of treatment, the untreated group still exhibited skin damage and inflammation, while the Blank-Gel group showed significant improvement in skin inflammation compared to the untreated group. The H-MnO_2_-Gel group, however, demonstrated the most noticeable recovery in skin damage (Fig. 5B, W5).

Dermatitis scores showed that all mice had a similar severity of the disease at the end of the 3rd week, and the scores decreased to varying degrees depending on the treatment (Fig. 5E). Compared to the untreated group, the Blank-Gel group showed a significant reduction in skin inflammation scores, while the H-MnO_2_-Gel group exhibited the lowest dermatitis scores. This suggests that H-MnO_2_ NPs and QCS/TA hydrogel have a synergistic effect in alleviating oxidative stress, leading to a significant improvement in skin inflammation. Epidermal thickness is an important indicator for evaluating AD. The untreated group and the Blank-Gel group had epidermal thicknesses that were 4.9 times and 3.6 times that of the healthy group, respectively. However, the H-MnO_2_-Gel group exhibited a significant restoration of epidermal thickness, measuring only 46% of the untreated group’s epidermal thickness (Fig. 5C, F). Due to the significant presence of a large number of mast cells in AD, we employed toluidine blue staining to observe mast cell infiltration. The results indicated that the group treated with H-MnO_2_-Gel exhibited the least infiltration of mast cells in the dermis of the skin (Fig. 5D, G).

**Fig. 6:**
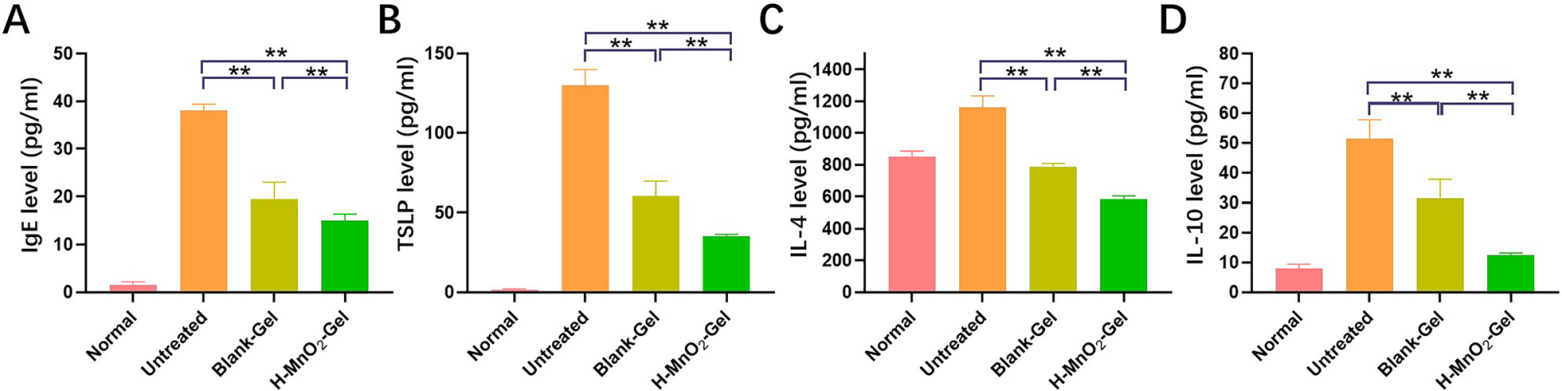
The concentrations of (**A**) IgE, (**B**) TSLP, (**C**) IL-4, and (**D**) IL-10 in blood serum retrieved from each group at W5.  (n = 6). **P<0.01.

Continuing our investigation, we conducted a further assessment of the alterations in immune proteins and cytokine levels associated with AD after the application of hydrogel treatment. Immunoglobulin E (IgE) serves as a representative biomarker in AD, known for its ability to heighten immune responses including mast cell activation and allergen internalization [27]. Remarkably, compared to the untreated group, we observed a noteworthy decrease in the elevated IgE levels within the blood of AD mice treated with H-MnO_2_-Gel (Fig. 6A). Keratinocytes can also secrete unique inflammatory chemokines and cytokines, among which thymic stromal lymphopoietin (TSLP) derived from keratinocytes plays an important role in AD. TSLP is undetectable in the normal or non-lesional skin of AD patients, but is highly expressed in acute and chronic AD lesions. Under the influence of TSLP, dendritic cells create a microenvironment that triggers the differentiation of inflammatory Th2 cells [28]. Research has also reported that TSLP can synergistically interact with IL-1 and TNFα to stimulate mast cells to produce high levels of Th2 cytokines. As shown in Fig. 6B, compared to the significant increase of TSLP in the untreated group, the Blank-Gel group showed a decrease of approximately 54% in TSLP levels. The H-MnO_2_-Gel group, on the other hand, exhibited a more pronounced reduction (approximately 73%).

ROS signaling participates in promoting IL-4 secretion after Th2 cell activation, and Th2 cells play a crucial role in the pathogenesis of AD, mainly leading to an increase in IL-4 levels [29]. Studies have shown that a decrease in intracellular ROS leads to a reduction in IL-4 secretion. Experimental results demonstrate that treatment with H-MnO_2_-Gel significantly reduces IL-4 levels, indicating that the clearance of ROS in the affected tissues also inhibits the Th2-mediated immune response (Fig. 6C). Additionally, ROS stimulates the production of IL-10 through calcium signaling pathways, which is involved in the generation of pro-inflammatory cytokines and T cell proliferation [30]. Inhibition of IL-10 has been found to contribute to AD alleviation. Application of H-MnO_2_-Gel on AD skin inhibits IL-10 levels **(**Fig. 6D**)**. In conclusion, treatment of AD with ROS-responsive H-MnO_2_-Gel can reduce the wound area on the skin of AD mice, restore epidermal thickness, and suppress AD-related immune factors, including mast cell infiltration, IgE, and inflammatory Th2 cytokines.

## Conclusion

Atopic dermatitis (AD) is a chronic and recurrent skin inflammation. During the pathogenesis of the disease, sustained high levels of ROS disrupt the balance of immune responses, exacerbating the condition of AD. In this study, a ROS-responsive hydrogel carrying hollow manganese dioxide nanoparticles (H-MnO_2_-Gel) was proposed to eliminate ROS, alleviate immune response imbalance, and regulate the damaged microenvironment. The hydrogel demonstrated a high efficiency in H_2_O_2_ scavenging, counteracting oxidative stress, and providing cell protection. In animal experiments, H-MnO_2_-Gel reduced skin thickness, mast cell count, IgE antibody levels, and inflammatory Th2 cytokines in AD-induced mice, promoting regeneration of inflamed tissues and enhancing the recovery of AD-affected skin. These findings suggest that the H-MnO_2_-Gel, by locally eliminating ROS and modulating the inflammatory microenvironment of skin damage, can improve the symptoms of AD and provide a novel strategy for the treatment and management of AD.

## Data Availability

This published article includes all data generated and analyzed during this research.
